# Nanoparticle targeting of de novo profibrotic macrophages mitigates lung fibrosis

**DOI:** 10.1073/pnas.2121098119

**Published:** 2022-04-04

**Authors:** Abhalaxmi Singh, Sreeparna Chakraborty, Sing Wan Wong, Nicole A. Hefner, Andrew Stuart, Abdul S. Qadir, Amitabha Mukhopadhyay, Kurt Bachmaier, Jae-Won Shin, Jalees Rehman, Asrar B. Malik

**Affiliations:** ^a^Department of Pharmacology and Regenerative Medicine, University of Illinois College of Medicine, Chicago, IL 60612;; ^b^Center for Lung and Vascular Biology, University of Illinois College of Medicine, Chicago, IL 60612;; ^c^Nano Biotherapeutics, Inc., Chicago, IL 60612;; ^d^Division of Cardiology, Department of Medicine, University of Illinois College of Medicine, Chicago, IL 60612;; ^e^Department of Biomedical Engineering, University of Illinois at Chicago, Chicago, IL 60612

**Keywords:** lung fibrosis, macrophages, mannosylation, nanoparticles, nanotherapeutics

## Abstract

The pathogenesis of lung fibrosis involves hyperactivation of innate and adaptive immune pathways that release inflammatory cytokines and growth factors such as tumor growth factor (TGF)β1 and induce aberrant extracellular matrix protein production. During the genesis of pulmonary fibrosis, resident alveolar macrophages are replaced by a population of newly arrived monocyte-derived interstitial macrophages that subsequently transition into alveolar macrophages (Mo-AMs). These transitioning cells initiate fibrosis by releasing profibrotic cytokines and remodeling the matrix. Here, we describe a strategy for leveraging the up-regulation of the mannose receptor CD206 in interstitial macrophages and Mo-AM to treat lung fibrosis. We engineered mannosylated albumin nanoparticles, which were found to be internalized by fibrogenic CD206^+^ monocyte derived macrophages (Mo-Macs). Mannosylated albumin nanoparticles incorporating TGFβ1 small-interfering RNA (siRNA) targeted the profibrotic subpopulation of CD206^+^ macrophages and prevented lung fibrosis. The findings point to the potential utility of mannosylated albumin nanoparticles in delivering TGFβ-siRNA into CD206^+^ profibrotic macrophages as an antilung fibrosis strategy.

## Significance

Current therapies for pulmonary fibrosis (PF) focus on slowing disease progression and reducing functional decline in patients by dampening the activation of fibroblasts and other implicated cells. There is a need for strategies that target the essential cells and signaling pathways involved in disease pathogenesis. Monocyte-derived macrophages (Mo-Macs) are known to express profibrotic genes and are involved in the pathogenesis of PF. Our results show that engineered mannosylated albumin nanoparticles specifically targeted disease-inducing Mo-Macs, and further, that nanoparticles efficiently delivered small-interfering RNA against profibrotic cytokine tumor growth factor β1 to prevent bleomycin-induced lung fibrosis.

Pulmonary fibrosis (PF) is a progressive devastating disease with few therapeutic options (1, 2). PF has a median survival of 2 to 5 y from time of diagnosis (3). COVID-19 survivors are also especially at risk due to the intersection of SARS-CoV-2–induced inflammatory lung injury (4) and activation of pathways triggering lung fibrosis (5–8). The medical burden of fibrotic lung disease is likely to increase due to an epidemic of chronic lung diseases resulting from environmental pollutants and climate change (5). PF pathogenesis involves the activation of innate and adaptive immune pathways releasing an array of growth factors and proinflammatory cytokines interleukin (IL)-4, IL-13, and tumor growth factor (TGF)β1 in response to which lung fibroblasts and macrophages acquire the profibrogenic phenotype and become resistant to apoptosis (9).

Recent evidence showed a crucial role of lung macrophages in disease pathogenesis through a profibrotic phenotype shift of these cells (10–13). The macrophage populations in lungs generated the cytokines TGFβ1, PDGFα, and VEGF (10) that promoted differentiation of fibroblasts into myofibroblasts and increased production of extracellular matrix (ECM) proteins (14, 15). It appears from these studies that specific lung macrophage populations are involved in the induction of fibrosis with complementary role played by lung fibroblasts, alveolar epithelial cells, and endothelial cells (12). Beyond the limited efficacy of dampening the activation of fibroblasts with drugs such as Pirfenidone or Nintedanib, there is no approach targeting the specific disease-inducing macrophage population (1). Lung transplantation is an option; however, donor scarcity severely limits its common usage (16).

While lung macrophages play a key role in the genesis of lung fibrosis (12, 17), the fundamental translational question whether a lung macrophage population mediating the disease can be targeted remains to be addressed. Studies showed that monocyte-derived alveolar macrophages (Mo-AMs) are a key effector cell in the pathogenesis of PF (11, 12). Monocyte-derived macrophages (Mo-Macs) take up residence in the lung interstitium where, on the basis of still ill-defined mechanisms related to the inflammatory niche, they express profibrotic mediators such as TGFβ1 (12). Deletion of these genes or ablation of Mo-Macs ameliorated lung fibrosis in mouse models (18). Mo-Macs in response to inflammatory cues also migrated into the lung airspace and transitioned into de novo AMs, referred to as Mo-AMs (12, 17). Transcriptomic and gene-expression analysis revealed that Mo-Macs displayed not only down-regulation of genes typically expressed in monocytes and up-regulation of genes expressed in AMs, but also markedly increased the expression of proinflammatory and profibrotic genes (11, 17, 19).

The question of the role of the transitioned Mo-Macs in disease pathogenesis and the feasibility of targeting of these cells remains unaddressed. To maximize the therapeutic potential of targeting macrophages, such an antifibrotic strategy would require the delivery to the pathogenic cell population while leaving intact the host-defense and tissue homeostatic functions of other lung macrophages. The concept bears resemblance to employing nanoparticles to target the monocytes and macrophages and delivering drugs to disrupt these disease-inducing cells in atherosclerotic tissue (20–23); there is precedence for this approach. Different nanoparticles have been designed with the aim of specifically targeting inflammatory macrophages in atherosclerotic plaques and also substantially improve the pharmacokinetic profile and chemical stability of nanoparticle-encapsulated therapeutics (20).

The ligands binding to the cell surface receptors by which macrophages are targeted include mannose, dectin, and phosphatidylserine (24). The expression of CD206 in AMs was shown to remain elevated during both acute and fibrotic phases in PF models (25). The number of CD206^+^ macrophages greatly increased during PF in both mouse models and patients (26–28), suggesting their therapeutic targeting may prove to be beneficial. In the present study, to target CD206^+^ macrophages, we engineered mannosylated albumin nanoparticles (MANPs) and showed that the Mo-AMs became the dominant lung macrophage population during fibrosis induced by bleomycin. Furthermore, delivering small-interfering RNAs (siRNA) against TGFβ1 via MANPs markedly mitigated fibrosis mediated by the activated population of Mo-Macs.

## Results

### Increased Profibrotic Mo-AM Population in Lung Fibrosis.

**Fig. 1.**
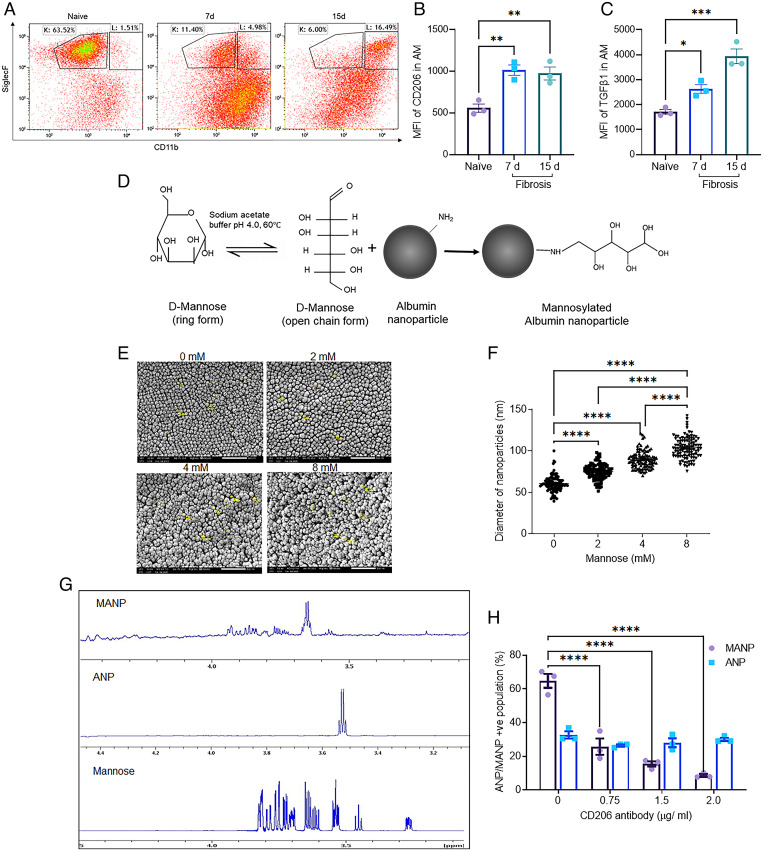
MANPs bind CD206^+^ macrophages. (*A*) Studies were made at baseline (naïve cells) and macrophages obtained from 7 d and 15 d postbleomycin-challenged mice as described in *Materials and Methods*. These times were chosen since this is window of maximum fibrosis in this model and this period is typically followed by reversal of the disease (17, 19). Results show changes in macrophage profile during development of PF. Tissue resident AM population (SiglecF^+^ CD11b^−^) seen in naïve control lungs decreased postbleomycin and this was coupled to the concominant appearance of a new population SiglecF^+^ CD11b^+^ AMs, referred to as Mo-AM. (Gating strategy: CD45^+^Gr1^−^CD64^+^). The gating strategy is described in *SI Appendix*, Fig. S1*C*. (*B*) CD206 expression was up-regulated in SiglecF^+^ AM after bleomycin-induced PF. AM in lung fibrosis acquired higher CD206 expression as compared to naïve AM. All SiglecF^+^ macrophages were analyzed (gating strategy: CD45^+^Gr1^−^CD64^+^SiglecF^+^). (*C*) TGFβ1 expression in AM was up-regulated during peak PF at day 7 and day 15. All SiglecF^+^ macrophages were analyzed (gating strategy: CD45^+^Gr1^−^CD64^+^SiglecF^+^). AM during lung fibrosis mice showed augmented TGFβ1 expression as compared AM in naïve mice paralleling the increase in CD206 (*B*). (*D*) Scheme for formulation of MANPs to be used in subsequent studies. The formulation involved first synthetizing naked ANPs and then conjugating mannose (as described in *Materials and Methods*). (*E* and *F*) SEM images showing the size and distribution of MANPs with different concentrations of mannose surface coating (Scale bar, 500 nm.) Changes in diameter of nanoparticles as a function of mannose coating as assessed by SEM. Nanoparticles (*n* = 100) in each condition were analyzed. (*G*) ^1^N NMR confirming the peaks of mannose in synthetized MANPs as compared to control ANP and free mannose. (*H*) Reduction in binding of CD206 to MANPs dependent on the concentration of anti-CD206 antibody. In contrast, this antibody had no effect on the interaction of CD206^+^ macrophages with ANP. These studies were made using BMDMs (*n* = 3). Statistical analysis by one-way ANOVA. *P < 0.05, **P < 0.01, ***P < 0.001 and ****P < 0.0001.

We carried out studies to address the shifts in lung macrophages during bleomycin-induced lung fibrosis. Bleomycin instillation induces PF (19, 29) during a 7-d time course experimental, which progresses up to the 15 d (*SI Appendix*, Fig. S1 *A* and *B*), as evident by time-dependent increase in lung tissue collagen deposition (*SI Appendix*, Fig. S1 *B*, *ii*), consistent with the known effects of bleomycin in modeling the disease (29, 30). To address the role of macrophages in disease pathogenesis, we determined changes in macrophage surface markers using flow cytometry (Fig. 1*A* and *SI Appendix*, Fig. S1*C*). Naïve lungs showed a population of tissue resident AMs positive for the AM surface marker (SiglecF^+^) and negative for inflammatory adhesion protein CD11b, as well as a population of interstitial macrophages (IMs) negative for SiglecF and positive for CD11b (Fig. 1*A*). Following bleomycin challenge Mo-Macs gradually acquired SiglecF expression and transitioned into Mo-AMs that were SiglecF^+^ and CD11b^+^ (Fig. 1*A*). The generation of the latter cells was coupled to a reduction in airspace resident AMs (SiglecF^+^ CD11b^−^) (Fig. 1*A*).

CD206 expression was also shown to markedly increase in AMs of idiopathic pulomonary fibrosis (IPF) patients and murine models of silicosis- and bleomycin-induced fibrosis (26, 27). On examining expression of CD206 in lungs of naïve and fibrotic mice, we observed elevated expression of CD206 in AMs of mice developing lung fibrosis at both 7 and 15 d postbleomycin exposure (Fig. 1*B*). We also observed in these cells elevated expression of the profibrotic mediator TGFβ1 at these times (Fig. 1*C* and *SI Appendix*, Fig. S1*D*). Examination of macrophage populations showed that SiglecF^+^ CD11b^+^ Mo-AMs displayed the highest expression of the cytokine TGFβ1 during fibrosis as compared to SiglecF low or naive resident AMs in lungs of unchallenged mice (*SI Appendix*, Fig. S1*E*).

### MANPs Target CD206^+^ Macrophages.

We next developed an approach to target the profibrotic macrophage population by leveraging the observed high surface expression of the mannose receptor CD206 (Fig. 1*B*). We coated albumin nanoparticles (ANPs) with mannose (Fig. 1*D*) because of its high binding affinity in the range of 1.0 to 2.0 nM to the mannose receptor CD206 (31). ANPs were synthesized by the coacervation process (32) and coated with D-mannose (Fig. 1*D*). Protein-based nanocarriers, such as ANPs, were used because of their safety profile and relevance to clinical translation (33). ANP synthesis using coacervation enabled high-efficiency encapsulation of molecules (34). Thus, we coated ANPs with D-mannose by opening the aldehyde groups at low pH and high temperature and then reacting the activated mannose with free amine groups of ANPs (35).

To optimize surface mannose concentration and produce uniform and saturated amounts of mannose coating, we studied the effects of increasing mannose up to 16 mM. Scanning electron microscopy (SEM) and mannose quantification showed that saturation occurred at 8-mM mannose (Fig. 1 *E* and *F* and *SI Appendix*, Fig. S2*A*). Upon coating the nanoparticles with mannose, the nanoparticle diameter increased from 60 ± 9 nm to 103 ± 12 nm (Fig. 1 *E* and *F*). Mannose quantification and ^1^NMR further showed that surface coating of mannose peaked between 3.5 to 4 ppm (Fig. 1*G*). To assess mannosylation stoichiometry, we determined molecular masses of ANP and MANP to be 1,218 and 1,429 MDa, respectively (*SI Appendix*, Fig. S2*B*). The hydrodynamic diameter of MANPs shifted from 120 ± 28 nm to 220 ± 28 nm after mannosylation with polydispersity index 0.3 (*SI Appendix*, Fig. S2*C*) and ζ-potential of −30 mV (*SI Appendix*, Fig. S2*C*). Thus, in all studies we used 220-nm mannosylated MANPs. The ability of MANPs to target CD206 was addressed using anti-CD206 blocking antibody, which significantly reduced MANP uptake in bone marrow-derived macrophages (BMDMs) in a concentration-dependent manner versus control ANPs (Fig. 1*H*).

### MANP Uptake by Profibrotic Macrophages.

**Fig. 2.**
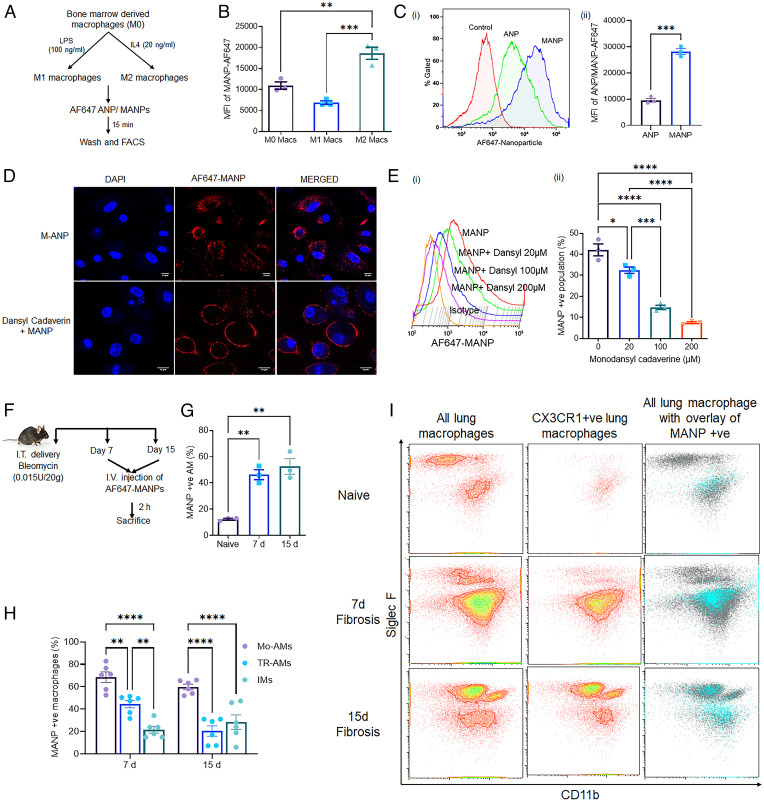
MANP uptake is dependent on the activation state of macrophages. (*A*) Schematic showing the generation of inflammmatory (M1) and antiinflammatory (M2) macrophages; studies were made using AF647 flurophore labeled MANPs to address interaction with BMDMs as described in *Materials and Methods*. (*B*) Internalization of AF647 dye-labeled MANPs by M0 (refers to basal untreated macrophages), M1, and M2 macrophages as determined by flow cytometry (*n* = 3). Uptake of MANPs in M2 macrophages was significantly greater than the other two groups of macrophages. (*C*) Internalization of MANPS by M2 macrophages was also significantly greater than uptake of nonmannosylated ANP by the same M2 macrophages (*n* = 3): (*i*) overlayed flowplots, (*ii*) quantification. (*D* and *E*) Monodansyl cadaverine, inhibitor of receptor-mediated endocytosis (56), in a concentration-dependent manner prevented MANP internalization by M2 macrophages as shown by confocal microscopy (*D*) and flow cytometry (*E*): (*i*) overlayed flowplots, (*ii*) quantifcation (*n* = 3). (*F*) Scheme of workflow to assess the distribution of AF647-labeled MANPs following bleomycin-induced lung fibrosis. (*G*) MANP^+^ SiglecF^+^ AM population in naïve unchallenged mice and in AMs during the zenith of lung fibrosis on day 7 and day 15 postbleomycin. MANPs were largely taken up by Mo-AMs (*n* = 3 animals for each group) and lung fibrosis significantly augmented the uptake of MANPs. (*H*) Comparison of MANP uptake by different macrophage populations after induction of PF (*n* = 6 animals). Comparisons were made among CD64^+^ SiglecF^+^ CD11b^−^ macrophages defined as long-term tissue resident AMs, SiglecF^+^ CD11b^+^ macrophages defined as newly arrived Mo-AMs, and SiglecF^−^ CD11b^+^ macrophages defined as Mo-IMs. MANPs at the 5- and 15-d peaks of fibrosis were most internalized by Mo-AMs and relatively fewer were internalized by resident AMs, as well as by transitional Mo-IMs. (*I*) Study showing uptake of MANPs by Cx3Cr1^+^ macrophages in Cx3cr1^CreERT2^ lineage mice. First panel (Gating strategy: CD45^+^Gr1^−^CD64^+^) representing all lung macrophages. Second panel (gating strategy: CD45^+^Cx3cr1^+^Gr1^−^CD64^+^) representing Cx3cr1^+^ monocyte-derived lung macrophages. Third panel shows overlay of all lung macrophages (gating strategy: CD45^+^Gr1^−^CD64^+^) (gray) and MANP^+^ macrophages (CD45^+^Gr1^−^CD64^+^MANP^+^) (cyan); results show association of MANP^+^ macrophages with Cx3cr1^+^ Mo-Macs. Statistical analysis was done by *t* test analysis and one-way ANOVA. *P < 0.05, **P < 0.01, ***P < 0.001, and ****P < 0.0001.

To test the uptake of MANP by CD206, we first carried out in vitro experiments using BMDMs. IL-4 was used to induce the transition of macrophages to antiinflammatory phenotype (classically referred to as M2) and up-regulate CD206, as opposed to LPS, which induced the transition to inflammatory macrophages (referred to as M1) (36). Fig. 2*A* shows the workflow of these experiments. We observed that internalization of AF647-labeled MANPs was greater in M2 macrophages than the control M0 or M1 macrophages (Fig. 2*B*). Internalization of MANPs by macrophages was also significantly augmented as compared nonmannosylated control ANPs in the IL-4–induced M2 macrophages (Fig. 2*C*). The increased uptake of MANPs by M2 macrophages was due to enhanced expression of the CD206 receptor (*SI Appendix*, Fig. S3*A*). Uptake via receptor-mediated endocytosis of MANPs was evident using monodansyl cadaverine, a clathrin-mediated endocytosis inhibitor (37), which prevented MANP internalization in a concentration-dependent manner (Fig. 2 *D* and *E*). The inhibition of nanoparticles from entering the cells by monodansyl cadaverine was confirmed using the membrane dye PKH-26 (*SI Appendix*, Fig. S3*B*).

As Mo-AMs are thought to be effector cells mediating PF (12, 17), we next assessed the distribution of MANPs among different lung macrophage populations to determine the macrophages optimally internalizing MANPs in vivo. In these studies, AF647-MANPs were intravenously injected in the following groups: naïve/control mice, 7-d fibrotic mice, and 15-d fibrotic mice. At 2 h post-MANP injection, flow cytometry was carried out in cells isolated from lungs of each group (Fig. 2*F*). We observed significant differences in internalization of MANPs by the SiglecF^+^ AM population before and after bleomycin (Fig. 2*G* and *SI Appendix*, Fig. S3*C*). Importantly, MANPs were seen to be preferentially internalized by CD11b^+^ SiglecF^+^ macrophages at both 7 d and 15 d postfibrosis induction (Fig. 2*H* and *SI Appendix*, Fig. S3*D*). This can be attributed to their CD206 expression pattern (*SI Appendix*, Fig. S3*E*). In quantifying MANP uptake postbleomycin, we observed that 65% of Mo-AMs internalized MANPs at day 7 as compared to 40% internalization by CD11b^−^ AMs and 20% by IMs (CD11b^+^ SiglecF^−^) (Fig. 2*H*). At day 15, MANP internalization by CD11b^+^ SiglecF^+^ Mo-AMs was also significantly greater (∼60%) vs. CD11b-AM and IM populations (20% and 25%, respectively) (Fig. 2*H*).

Next, to confirm that MANP^+^ macrophages were indeed monocyte-derived, we carried out internalization studies in Cx3cr1Cre^ERT2+^, Tdtomato^fl/fl^ genetic lineage tracing mice, in which circulating monocytes are Tdtomato^+^. In these studies, we observed ∼75% MANP^+^ macrophages were SiglecF^+^ Cx3cr1^+^ Mo-AM (Fig. 2*I*), indicating that Mo-AMs were largely taking up MANPs. Furthermore, the Cx3cr1^+^ macrophage population that were MANP^+^ were present in the vicinity of Pdgfrb^+^ fibroblasts (*SI Appendix*, Fig. S4*A*). This observation is consistent with the fibrogenic role of Cx3cr1^+^ SiglecF^+^ macrophage clustered in proximity to fibroblasts defined as Pdgfrb^+^ (17).

### Functional Heterogeneity of AMs Internalizing MANP.

**Fig. 3.**
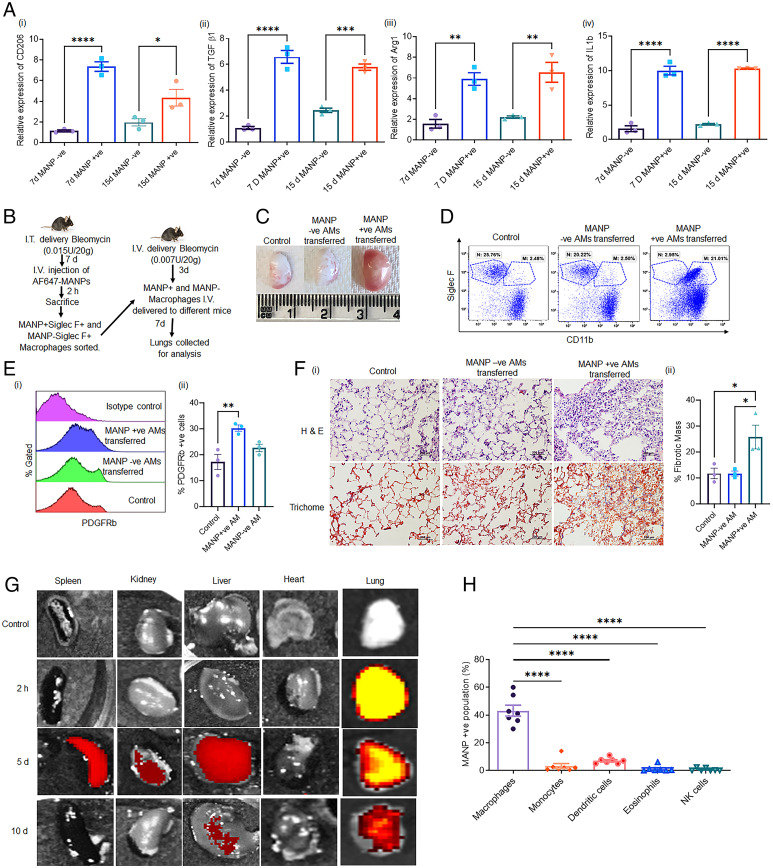
Uptake of MANPs by CD206^+^ Mo-Macs in lungs and transfer of fibrosis induced by CD206^+^ macrophage injection into naïve lungs. (*A*) Uptake of MANPs is associated with expression of CD206, TGFβ1, Arg1, and IL-1β at both 7 d and 15 d postbleomycin-induced lung fibrosis. Results show quantitative PCR differences in relative mRNA expression in MANP^+^ AMs vs. MANP^−^ AMs at 7 d or 15 d after induction of fibrosis using bleomycin; (*i*) CD206, (*ii*) TGFβ1, (*iii*) Arg1, (*iv*) IL-1β. Fold-changes in expression were calculated relative to 18S rRNA gene and determined by comparative Ct method. Data points represent results from individual mice (*n* = 3 mice). (*B*) Scheme for the adoptive transfer experiment used to address effects of transferring MANP^+^ AMs and MANP^−^ AMs into naïve primed lungs. (*C*) Lung images showing differences between control mice, MANP^−^ AM transferred mice, and MANP^+^ AM transferred mice. Fibrosis was only induced by transferring MANP^+^ AM into naïve lungs. (*D*) Flow cytometry dot-plot representing profiles of lung macrophages (CD45^+^Gr1^−^CD64^+^) in control mice, MANP^−^ AM-injected mice, and MANP^+^ AM-injected mice. Results show that MANP^+^ AM transferred mice induced depletion of tissue resident AM and concomitant influx of Mo-AM. (*E*) Pdgfrb^+^ fibroblasts (CD45^−^ Pdgfrb^+^) in different groups showing relative number of fibroblasts in control, MANP^−^-transferred mice, and MANP^+^-transferred mice: (*i*) overlayed flowplots, (*ii*) quantification from three independent experiments. Fibroblasts were associated to a greater extent with the MANP^+^ AM population. (*F*, *i*) Histological images of lungs using H&E and Trichome staining in control group, MANP^−^ AM-transferred group, and MANP^+^ AM-transferred group. Transfer of MANP^+^ AM-induced lung fibrosis in recipient mice in contrast to MANP^−^ AM indicating that MANP^+^ AM (which are also CD206^+^, TGFβ1^+^, Arg1^+^, and IL-1β^+^) recapitulate fibrosis in naïve mice. (*ii*) Quantification of percentage of fibrotic mass from three independent experiments. Quantification was done using ImageJ. (*G*) In vivo imaging using in vivo imaging system (IVIS) of different organs at 2 h, 5 d, and 10 d postinjection of AF647-MANPs showing the highest localization in lungs. Besides lungs where MANPs persisted up to 10 d postbleomycin (time of maximum fibrosis), the liver and spleen also showed uptake of MANPs but only at 5 d indicating spillover of MANPs into these peripheral organs. (*H*) In vivo distribution of AF647-MANPs in lung immune cells in 7 d lung fibrotic mice (*n* = 7 animals). The gating strategy of immune cells was following: macrophages = CD45^+^Gr1^−^CD64^+^; monocytes = CD45^+^Ly6G^−^MHCII^−^CD64^+^CD11b^+^; dendritic cells: CD45^+^Ly6G^−^CD64^−^MHCII^+^; eosinophils: CD1b^−^CD45^+^Ly6G^−^CD64^−^MHCII^−^CD11b^+^; NK cells = CD45^+^Ly6G^−^MHCII^−^CD64^−^CD11b^+^. These results showed highest uptake of MANPs by Mo-IM and Mo-AM. Statistical analysis was carried out by One-way ANOVA. *P < 0.05, **P < 0.01, ***P < 0.001, and ****P < 0.0001.

To assess the phenotype of macrophages internalizing MANPs, we sorted MANP^+^ SiglecF^+^ AMs and MANP^−^ SiglecF^+^ AMs and determined expression of profibrotic and proinflammatory genes. MANP^+^ macrophages showed significantly greater expression of the mannose receptor CD206, consistent with the role MANP uptake by CD206^+^ macrophages (Fig. 3 *A*, *i*). We also observed significant increases in the expression of profibrotic mediators TGFβ1, IL-1β, and Arg1 (27) on both day 7 and day 15 in the MANP^+^ Mo-AMs, (Fig. 3*A*).

We next carried out an adoptive transfer experiment to address whether MANP^+^ Mo-AMs could induce PF in low dose bleomycin primed mice. MANP^+^ Mo-AMs obtained from fibrotic mice were sorted as described in *Materials and Methods*, and intravenously injected in naïve mice prechallenged with low priming dose of bleomycin to provide the requisite inflammatory milieu (Fig. 3*B*). Engraftment of Mo-AMs was confirmed by labeling the cells with 5-(and 6)-Carboxyfluorescein diacetate succinimidyl ester (CFSE) dye, which also allowed cell tracking 3 d after injection (*SI Appendix*, Fig. S4*B*). Transfer of MANP^+^ Mo-AMs significantly augmented the development of fibrosis in primed lungs (Fig. 3*C*) and also induced influx of CD11b^+^ Mo-AMs in these mice (Fig. 3*D* and *SI Appendix*, Fig. S4*C*). Lung expression of Pdgfrb, a marker of fibroblasts, was also increased (Fig. 3*E*), and associated with histological changes indicative of fibrosis in MANP^+^ cell transfer mice (Fig. 3*F*).

### Profibrotic Macrophages Internalize MANP.

We observed that the injected AF647-labeled MANPs were largely found in lungs for up to 5 d after injection of MANPs (Fig. 3*G*), whereas there was minimal MANP signal in the spleen, kidneys, and liver at this time. The lung signal persisted up to 10 d although at diminishing levels (Fig. 3*G*). We also studied the distribution profile of MANPs in different cell types and observed that MANPs were preferentially internalized by macrophages as compared to other immune cells (Fig. 3*H*). At postfibrosis day 7, 60% of macrophages had internalized MANPs whereas internalization by monocytes was only 2% indicating that monocytes only internalized MANPs after entering the lungs of mice and transitioning into Mo-AMs. Furthermore, there was negligible internalization of MANPs by dendritic cells, eosinophils, and natural killer (NK) cells (Fig. 3*H*).

### TGFβ1 siRNA Delivery via MANP Prevents Bleomycin-Induced Lung Fibrosis.

**Fig. 4.**
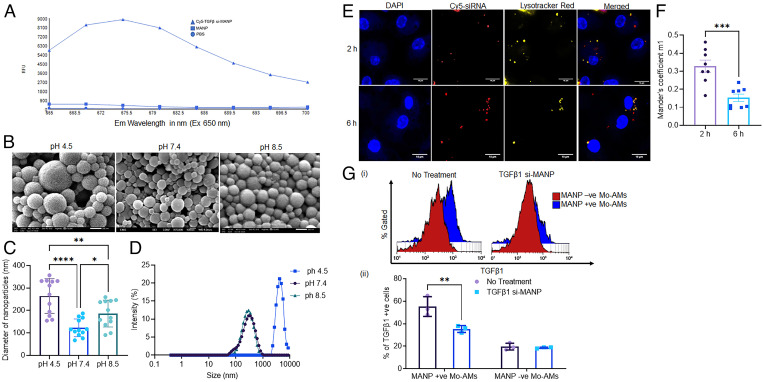
TGFβ1 siRNA incorporated in MANPs targets fibrogenic Mo-AM. (*A*) Emission spectrum of Cy5-TGFβ1 siRNA MANPs showing entrapment of siRNA in MANP. (*B–D*) SEM of MANPs at different pH conditions for 3 h. Diameter of MANPs at different pH values imaged by SEM. Hydrodynamic size of MANPs measured by dynamic light scattering at pH 7.4, 4.5, and 8.5. (Scale bars in *B*, 200 nm for pH 4.5 and pH 8.5, and 100 nm for pH 7.4.) (*E* and *F*) Confocal microscopy images showing escape of Cy5-labeled TGFβ1 siRNA from MANPs and lysosomes within 6 h of posttreatment. Results show release of Cy5 from MANPs at 6 h. (*F*) Mander’s colocalization coefficient calculated from confocal microscopy images from *E*; this indicates the fraction of Cy5-siRNA overlapping with Lysotracker red, indicating initial high localization of TGFβ1 siRNA in lysosomes and gradual reduction with time. (*G*) Expression of TGFβ1 in MANP^+^ and MANP^−^ Mo-AMs before and after treatment of mice with TGFβ1 siRNA-MANPs. Treatment was carried out on day 3 postbleomycin induction of fibrosis and mice were killed on day 7. (*i*) Histoplot, (*ii*) quantification (*n* = 3 animals). Statistical analysis was carried out by one-way ANOVA. *P < 0.05, **P < 0.01, ***P < 0.001, and ****P < 0.0001.

Profibrotic macrophages release TGFβ1 to activate fibrogenic signaling pathways (10). Macrophages are also a source of tissue inhibitors of metalloproteinases antagonizing MMP-mediated ECM degradation and remodeling (10). We synthetized MANPs incorporating TGFβ1 siRNA to neutralize the TGFβ1 generated by the CD206^+^ Mo-AMs. The loading of TGFβ1 siRNA was evident by fluorescence measurements using Cy5-labeled siRNA (λ maximum absorption [650 nm], λ maximum emission [675 nm]) (Fig. 4*A*). The entrapment efficiency of siRNA was ∼63% and in PBS at 37 °C 50% of the siRNA was released in 5 d (*SI Appendix*, Fig. S5*A*). Evaluating internalization of Cy5-TGFβ1-siRNA-MANP in vitro in M0, M1, and M2 macrophages, we found by far the most significant internalization occurred in M2 macrophages (*SI Appendix*, Fig. S5*B*). On comparing internalization of unencapsulated siRNA and siRNA-MANP, we observed little internalization in the former as compared to the latter (*SI Appendix*, Fig. S5*C*).

We next studied the behavior of nanoparticles at different pH values of 4.5, 7.4, and 8.5 after 3 h of incubation, and observed that pH of 4.5 increased nanoparticle size (Fig. 4 *B* and *C*). Similar results were obtained using dynamic light scattering. The diameter was similar at pH of 7.4 and 9.0, whereas it increased by 10-fold at pH 4.5 (Fig. 4*D*). Overnight incubation of nanoparticles at pH 4.5, 7.4, and 8.5 showed that they were stable only under neutral and alkaline conditions (*SI Appendix*, Fig. S2*D*), suggesting more probable cargo escape from nanoparticles after entry into the acidic endo-lysosomal complex (Fig. 4*D*).

To address delivery of siRNA into cells, we studied the localization of Cy5-labeled siRNA by confocal microscopy (Fig. 4*E*). At the initial 2-h time point, most siRNA incorporating-nanoparticles was colocalized with the Lysotracker signal, whereas siRNA was released into the cytoplasm within 6 h (Fig. 4*E*). The presence of siRNA was also observed up to 24 h postincubation (*SI Appendix*, Fig. S4*D*). The overlapping of Lysotracker signal with the Cy5 signal was greater at 6 h than 2 h, attributable to endocytosis of MANPs relative to their escape from lysosomes as was also seen during siRNA delivery by nanoparticles in breast cancer (38). Mander’s coefficient calculated from confocal images showed gradual decrease in overlapping of signals from Cy5-siRNA with Lysotracker (Fig. 4*F*), providing the optimal window for assessing the efficacy of MANP therapy.

We next evaluated TGFβ1 expression by flow cytometry in nanoparticle positive and negative AMs. We observed significantly reduced TGFβ1 expression in AMs of the treatment group as compared to control group (Fig. 4*G*). TGFβ1 expression due to siRNA delivery was reduced only in the nanoparticle-internalizing cells (Fig. 4*G*), indicating the specificity of MANP targeting.

**Fig. 5.**
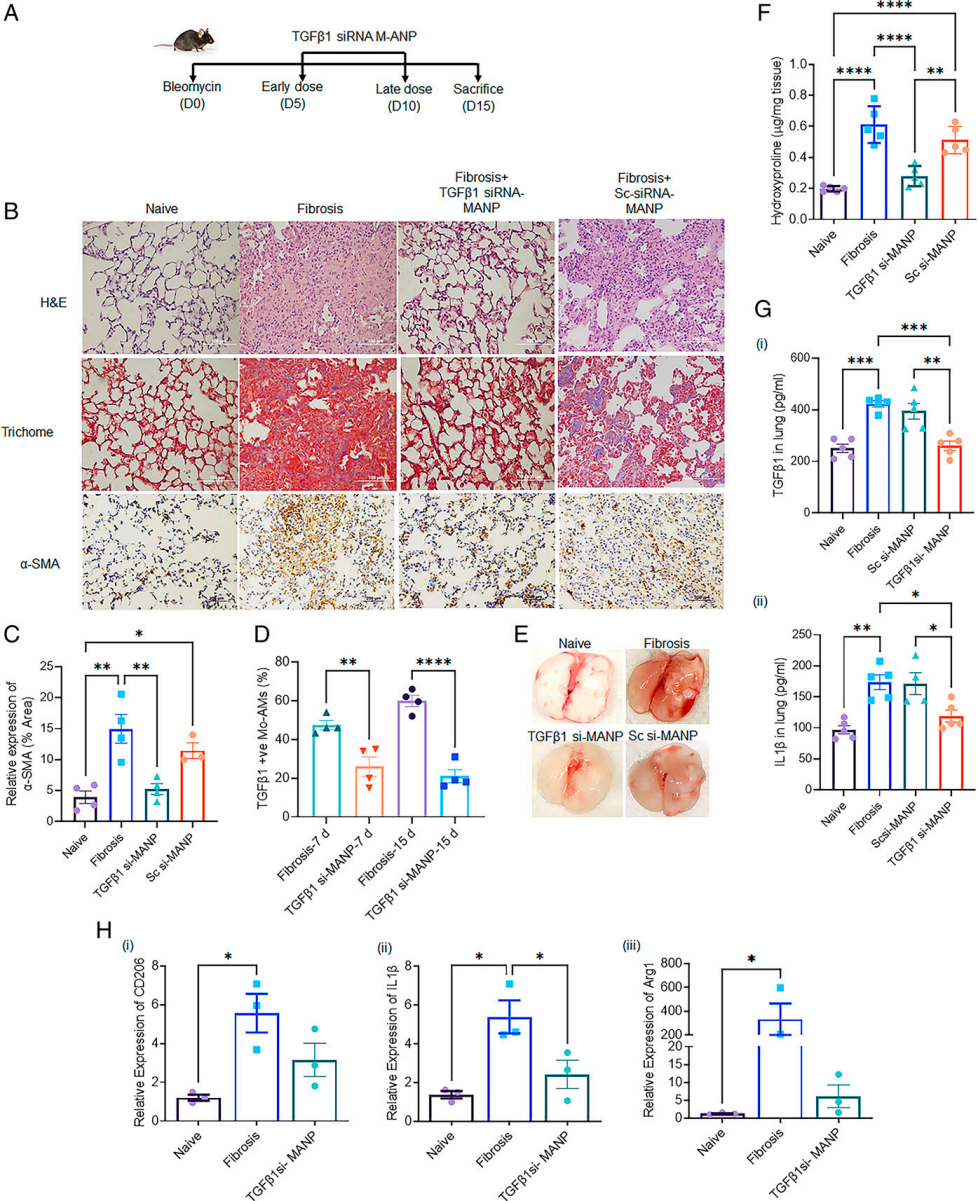
TGFβ1 siRNA incorporated in MANPs mitigates lung fibrosis. (*A*) Scheme of workflow for therapeutic evaluation of TGFβ1 siRNA-MANPs. (*B*) Histology of lungs from naïve mice, mice at day 15 postfibrosis induced by bleomycin, and MANP (scrambled and TGFβ1 siRNA) -treated mice at day 15 postfibrosis induced by bleomycin. Tissues were assessed by H&E, Trichome staining and α-SMA staining. (*C*) Quantification of relative expression of α-SMA from histological images in independent experiments. Quantification was made using ImageJ. (*D*) Expression of TGFβ1 in Mo-AM after 7 d and 15 d postfibrosis with and without treatment with siRNA-MANP. (*E*) Micrographs show lung fibrosis induced by bleomycin at day 15 as compared to naïve mice and mice treated TGFβ1-siRNA-MANPs or TGFβ1 scrambled siRNA-MANP–treated mice. (*F*) Hydroxyproline assay showing collagen content in lungs of naïve, 15 d postfibrosis, 15 d TGFβ1-siRNA-MANP treated and 15 d scrambled siRNA-MANP treated mice. (*G*) ELISA showing levels of cytokines of (*i*) TGFβ1 and (*ii*) IL-1β in lung tissue homogenates. (*H*) mRNA expression of (*i*) CD206, (*ii*) IL-1β, and (*iii*) Arg1 in naïve, 15-d fibrotic mice, and 15-d fibrotic TGFβ1 siRNA-MANPs–treated mice. Fold-change in expression was calculated relative to 18S rRNA gene, determined by comparative Ct method. Each data point represents results from macrophages pooled from two mice. Statistical analysis was carried out by one-way ANOVA. *P < 0.05, **P < 0.01, ***P < 0.001, and ****P < 0.0001.

We next evaluated the effects of TGFβ1 siRNA-loaded MANPs on bleomycin-induced lung fibrosis for up to 15 d. TGFβ1 siRNA-MANPs were injected at day 5 and day 10 after bleomycin instillation to target the infiltrating Mo-AMs (Fig. 5*A*). We observed marked reduction in fibrosis after treatment with TGFβ1-siRNA-MANPs from histological analysis including Trichome staining and α-smooth muscle actin (α-SMA) levels (Fig. 5 *B* and *C* and *SI Appendix*, Fig. S6*A*). Significant reductions were also seen in TGFβ1 expression in Mo-AMs obtained after treatment with TGFβ1-siRNA-MANPs in the 7-d and 15-d lung fibrosis mice (Fig. 5*D*). We also observed reductions in fibrotic mass after treatment (Fig. 5*E*). Hydroxyproline assay also showed significant reduction after treatment (Fig. 5*F*) indicative of reduced collagen deposition. To study the effects of the treatment on proliferation of macrophages, we performed the BrdU assay, and observed that 6% of all AMs were in the proliferative state in control lungs, whereas AM proliferation doubled in fibrotic lungs (*SI Appendix*, Fig. S6*B*). Proliferation was however reduced by threefold in TGFβ1-siRNA-MANP–treated mice (*SI Appendix*, Fig. S6*B*).

We further assessed the concentrations of profibrotic cytokines TGFβ1 and IL-1β in lungs by ELISA. Treatment with TGFβ1-siRNA-MANPs reduced cytokine generation (Fig. 5*G*). To assess the fate of macrophages in vivo, we sorted AMs from naïve, fibrotic, and treated mice and performed quantitative PCR. Treatment with TGFβ1-siRNA-MANPs reduced the expression of the profibrotic markers CD206, IL-1β, and Arg1 (Fig. 5*H*).

**Fig. 6.**
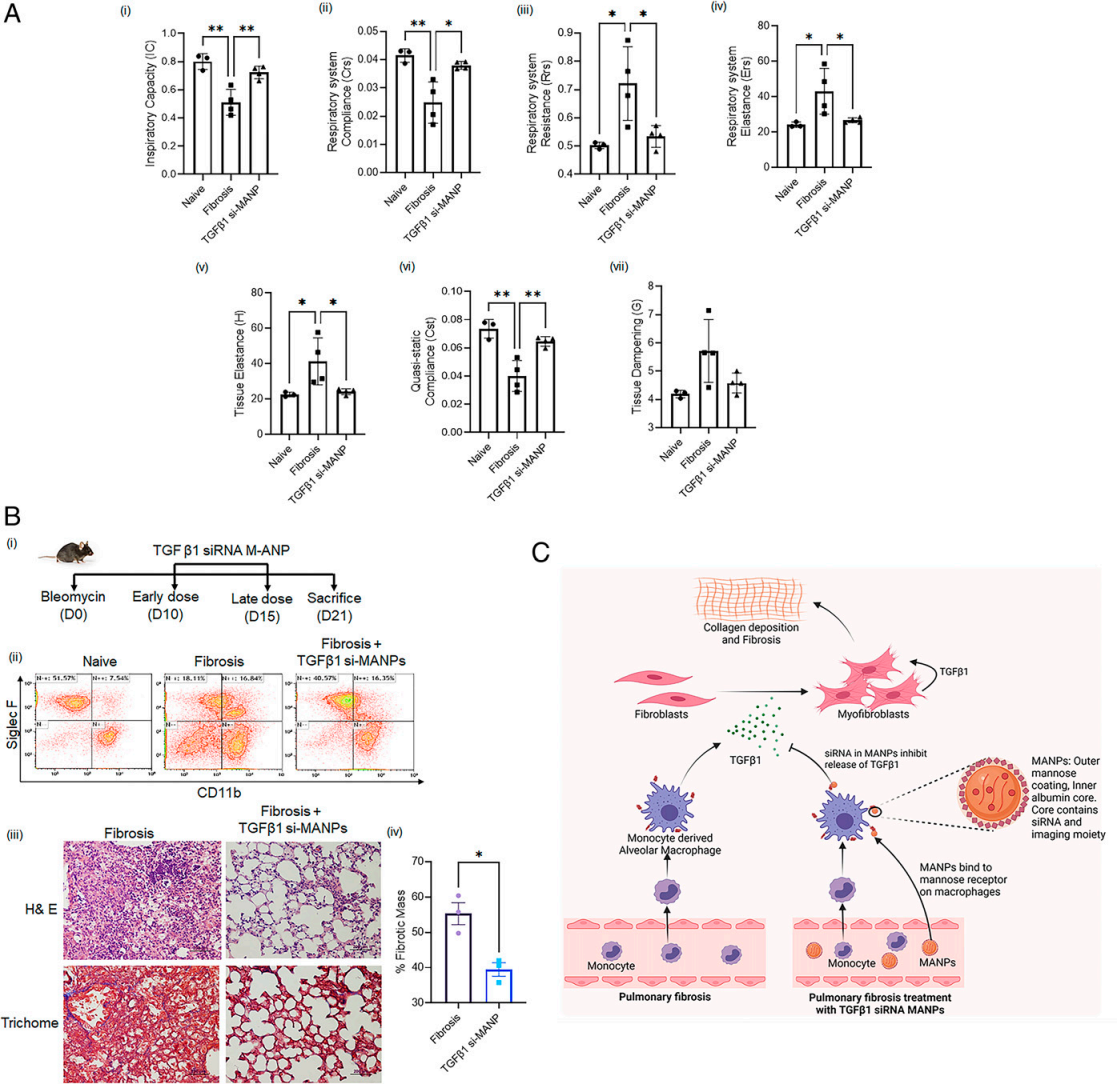
TGFβ1 siRNA incorporation in MANPs restores lung function and reverses fibrosis. (*A*) Lung function determination using flexiVent system on 15 d after bleomycin-induced PF. (*i*) Inspiratory capacity, (*ii*) respiratory system compliance, (*iii*) respiratory system resistance, (*iv*) respiratory system elastance, (*v*) tissue elastance, (*vi*) quasi-static compliance, and (*vii*) tissue dampening. Dots in plot shows values obtained from individual mice. (*B*,*i*) Schematic of treatment schedule for 21day fibrosis mice. (*ii*) Changes in macrophage profile after treatment at 21 d of fibrosis. (*iii*) Histological evaluation by H&E staining and Trichome staining to detect therapeutic effect. (*iv*) Quantification of percent fibrotic mass from Trichome staining of histology samples from three independent experiments. The quantification was done using ImageJ. (*C*) Scheme representation showing actions of MANPs in targeting the fibrosis-inducing Mo-AMs population. Induction of fibrosis involves Mo-AM–mediated release of profibrotic cytokines that activate endothelial cells and fibroblasts toward profibrotic phenotype leading to fibrosis (10). We demonstrated that MANPs targeting of profibrotic Mo-AMs since using TGFβ1 siRNA incorporating MANPs mitigated fibrosis. Statistical analysis was carried out by one-way ANOVA. *P < 0.05; **P < 0.01.

Next, we tested therapeutic efficacy of TGFβ1-siRNA-MANPs by measuring lung function. We measured multiple mechanical properties (inspiratory capacity, tissue elastance, respiratory system resistance, respiratory system compliance, respiratory system elastance, quasi-static compliance, and tissue dampening) of the respiratory system using the flexiVent instrument. We observed significant improvements in respiratory mechanics after treatment with MANPs loaded with TGFβ1 siRNA (Fig. 6*A*). Next to test the effects TGFβ1-siRNA-MANP in a model of established lung fibrosis, we assessed a different treatment regime. We injected TGFβ1-siRNA-MANPs on day 10 and day 15 and studied the severity of the disease on day 21 (Fig. 6 *B*, *i*). Macrophage profiling (Fig. 6 *B*, *ii*) and histological analysis (Fig. 6 *B*, *iii*, *iv*) at these times after injection of therapeutic MANPs showed reversal of the disease-based MANP-mediated delivery of siRNAs into monocyte-derived macrophages, as described in model figure Fig. 6*C*.

## Discussion

Fibrosis affects multiple organs and accounts for ∼45% of all deaths in the industrialized world, and more so in the rapidly developing economies (39). There are about 100,000 patients in the United States with idiopathic PF and up to 40,000 new cases each year (40). Multiple etiologic factors have been proposed but there remains a translational chasm in effective treatments (39). It is increasingly recognized that aberrant activation of profibrotic monocyte-derived alveolar macrophages (SiglecF^+^ CD11b^+^ Mo-AMs) and complex interaction between these lung macrophages and fibroblasts is a major determinant of the disease pathogenesis (11, 12, 17, 41). Studies in mice in which circulating monocytes were depleted using clodronate showed significantly reduced fibrosis (42). Several studies showed that depletion of Mo-AMs and reduced release of profibrotic mediators also prevented lung fibrosis (4, 11, 26, 27). There appears to be a distinction between newly arrived Mo-AMs and tissue resident AMs of embryonic origin in disease pathogenesis in that latter do not appear to have a significant role in fibrosis (11, 12). Studies showed that fibrosis onset in experimental models resulted in time-dependent depletion of the resident AMs and the influx of Mo-AMs originating from extravasation of monocytes and their migration into the airspace (43).

Single-cell RNA sequencing has previously identified the gene-expression profiles of Mo-AMs as CX3CR1^+^ SiglecF^+^ (17). Mo-AMs localized in the fibrotic niche are believed to induce the disease through unchecked release of fibrogenic cytokines, such as TGFβ1 (17, 43). We report here that an ANP-based approach prevented bleomycin-induced lung fibrosis. We demonstrated: 1) a process for formulating MANPs that efficiently encapsulated the antifibrotic siRNA against TGFβ1, 2) preferential targeting of MANPs into profibrotic Mo-AMs (CX3CR1^+^ SiglecF^+^ population) that supported the key role of these cells as drivers of lung fibrosis, and 3) delivery of TGFβ siRNA via MANPs to the profibrotic macrophage population in mitigating the fibrosis burden.

We used the widely employed bleomycin model of lung fibrosis. The method is useful in that the disease severity and time course are well characterized, thus providing an assessment of treatment efficacy (19, 44). The primary concerns with the model are: 1) its relevance, as there is an initial phase of lung inflammation induced by bleomycin, which regresses within 3 d, and precedes the fibrogenic phase (19); and 2) the self-limiting nature of fibrosis typically terminating at 25-d postbleomycin insufflation (19, 44). To test the efficacy of MANP, studies were made during the period of florid fibrosis (and not during the early inflammatory phase) and time course results were compared using control scrambled siRNA-loaded MANPs having the same characteristics such as diameter and electrostatic charge as MANPs.

Various nanoparticle strategies have been used to modulate lung fibrosis. These include cerium oxide nanoparticle delivery of microRNA-146a for treatment of acute lung injury and fibrosis (45). PEI nanoparticles incorporating GSE4 were delivered via airway postbleomycin, which increased telomerase activity and decreased DNA damage and showed antifibrotic activity (46). A liposomal approach has also been used to target a profibrotic macrophage population by depleting the cell-surface methyl-CpG–binding domain 2 (MBD2) (4). However, liposomal formulations can be internalized nonspecifically by any macrophage. Our approach using MANP was cell-specific in targeting the fibrogenic CX3CR1^+^ SiglecF^+^ population of macrophages expressing the mannose receptor CD206. Using this strategy, we identified a more efficient approach to induce disease regression using MANPs to deliver TGFβ1 siRNA into the profibrotic monocyte-derived population.

A key element of the studies was leveraging the observed enhanced expression of the mannose receptor CD206^+^ in profibrotic Mo-Macs to target these cells using mannosylated nanoparticles. Mo-Macs became the abundant cell type in airways on replacing the resident long-term AMs following induction of fibrosis. We demonstrated that the engineered MANPs bound the mannose receptor CD206 in Mo-Macs. On comparing the uptake of MANPs in SiglecF^+^ CD11b^−^ resident AMs vs. SiglecF^+^ CD11b^+^ Mo-AMs, we observed significantly greater internalization in the Mo-AMs. The greater uptake may be attributed to injured endothelial barrier during bleomycin-induced lung inflammation and fibrosis that facilitates the entry of intravenously injected nanoparticles permitting monocyte extravasation into the lung interstitium (47). Importantly, the high CD206 expressing Mo-Macs also expressed the profibrotic cytokine TGFβ1. Injection of MANPs incorporating TGFβ1-siRNA (TGFβ-siRNA-MANPs) 5 d after the induction of bleomycin-induced fibrosis significantly reduced disease severity. These results provide proof-of-concept that targeting the monocyte-derived macrophage population mediating lung fibrosis reversed the course of the disease.

MANPs loaded with siRNA have the potential of delivering siRNA by evading rapid blood clearance via nuclease degradation and through poor endosomal escape of the siRNA (38, 48). While mannosylated nanoparticles preferentially target profibrotic Mo-Macs and delivered the construct into cells to mitigate fibrosis, we observed that targeting these cells did not appear to disrupt the host-defense function of macrophages. Because of the specific targeting of CX3CR1^+^ SiglecF^+^ with MANPs, other cells continued to release of essential host-defense cytokines IL-1β and IL-6.

Temporal analysis of lung tissue responses have previously been shown to demonstrate an immediate increase in inflammation and number of Mo-AMs at day 3 and a decline after 14 d postbleomycin instillation (19). In studies in which fibrosis was established for up 10 d postbleomycin, we assessed whether the disease could be reversed with TGFβ-siRNA-MANP treatment. We observed reversal of fibrosis when injection was made on day 10 and day 15 postbleomycin induction of disease. These results show promise through targeting of the lung fibrosis-inducing population of Mo-Macs and Mo-AMs by TGFβ-siRNA-MANPs delivered via the intravenous route. This was possible since lung fibrosis is accompanied by lung vascular injury (leakiness of the endothelial barrier) which persists in case of progressive fibrotic diseases as in idiopathic PF (47, 49). Thus, the intravenous delivery route is feasible due to the injury-induced leakiness of the lung vascular barrier, which allows for the entry of the nanoparticles into the tissue. However, airway delivery was unsuccessful, suggesting the need to target the activated Mo-Macs and the transitioning Mo-AMs prior to the cells entering the airspace. Thus, intravenous MANPs enabled targeting of the population of profibrotic macrophages and delivery of TGFβ1 siRNA to prevent lung fibrosis and improve survival.

The mechanisms of MANP-induced reversal of lung fibrosis are likely multifaceted. Our results showed that MANP target CX3CR1^+^ SiglecF^+^ macrophages localized in the inflammatory and fibrotic niche in vivo. The protection may thus be the result of disruption of signaling interaction between the targeted CX3CR1^+^ SiglecF^+^ macrophages and dampening of the proximal fibrogenic fibroblasts by counteracting dysregulated transcriptional programs in fibroblasts (50). Other possibilities could involve the role of CX3CR1^+^ SiglecF^+^ macrophages in epithelial–mesenchymal transition believed to contribute to lung fibrosis (51) and the inhibition of NF-κB–mediated profibrotic gene expression induced by CD148 phosphatase in fibroblasts (52). Finally, the MANP-targeted macrophages may inactivate Fas signaling that leads to fibroblast survival and persistence and continued profibrotic functions of lung fibroblasts (53).

In summary, we demonstrate the therapeutic efficacy of using mannosylated nanoparticles to target a specific subset of profibrotic lung macrophages and thus mitigate lung fibrosis.

## Materials and Methods

### Preparation of MANP.

The ANPs were synthesized following a desolvation technique using Bovine Serum Albumin (BSA, molecular mass 66,000 Da; Sigma) with modification to an earlier protocol (32). BSA was diluted in endotoxin-free water (20 mg/mL) and reacted with ethanol (3.5 mL) with gradual addition over a period of 10 min. The formed nanoparticles were stabilized by adding 38 µL glutaraldehyde (25%; Sigma) and allowed to stir overnight. To encapsulate Alexa fluor 647 or Cy5 modified TGFβ-siRNA, the dye in 25 µL DMSO or the siRNA in 100 µL 5% DMSO was first thoroughly mixed with the BSA solution by stirring for 1 h before proceeding to reaction with ethanol. ANPs were collected by centrifugation (15,000 × *g*, 20 min, 4 °C.) and washed three times by resuspension in endotoxin-free water (1 mL). After the third wash, the pellet was resuspended in high concentration (∼20 g/mL).

To mannosylate the ANPs, D-mannose (Sigma) was dissolved in sodium acetate buffer (pH 4.5) and heated at 60 °C for 1 h to open the mannose ring and expose the aldehyde group for further conjugation process. Then, the cooled down activated mannose was added to the ANP solution (1:5 [vol:vol] ratio) in endotoxin-free water and left for overnight stirring, and next day the MANPs were collected by centrifugation (15,000 × *g*, 20 min, 4 °C.) and washed three times by resuspension in endotoxin-free water (1 mL). After the third wash, the pellet was resuspended in PBS. The release kinetics of the Cy5-TGFβ1 siRNA from the MANPs were analyzed in PBS at 37 °C by a fluorescence spectrophotometer.

### Characterization of MANPs.

The physical characterization of nanoparticles was performed by measuring the hydrodynamic size and the surface ζ-potential using Malvern Zeta sizer. The SEM of the uncoated and different concentration mannose-coated nanoparticles were done by drying 2 mg of nanoparticles and then gold sputter-coated before viewing them under JEOL JSM-IT500HR SEM. The mannose amount in the nanoparticles was quantified using a mannose quantitation kit (Megazyme).

Standard proton NMR techniques were utilized to confirm the conjugation of the mannose to BSA nanoparticles. All experiments were conducted at 298 K on a Bruker 600 MHz AVANCE III NMR spectrometer operating with a 5 mm D-CH cryogenic probe equipped with a *z*-axis pulsed field gradient. One-dimensional (1D) proton spectra were acquired using the standard excitation sculpting 1D water suppression experiment with a total of 256 scans, a sweep width of 16 ppm, acquisition time of 2 s, and a 2-s relaxation delay. Calibration of the proton 90° pulse was verified by null signal of a 360° pulse. Spectra were processed with manual phasing and peak integration.

To quantify the molecular mass of the uncoated and mannosylated nanoparticles, we used ultracentrifugation to obtain a stoichiometric view of the mannose per nanoparticle. The samples were dispersed in 1× PBS (46-013-CM from Corning) and the same PBS was used as reference buffer during the SV runs. The aliquots of sample were equilibrated to room temperature before loading to standard centrifugation cells with Epon centerpiece and sapphire windows with 1.2-cm path length. We used 400 µL of PBS and 420 µL of samples to load into each sector of the cells. The cells were then placed in an An60-Ti rotor which was then placed in the centrifuge under vacuum at 20 °C and subjected to SV at 35,000 rpm for BSA, 3,000 rpm for ANP, and 1,000 rpm for the mannose-ANP, respectively. Absorbance data at 280 nm were acquired. The data were analyzed using Continuous c(S) distribution methodology fitted with Lamm equations in the SEDFIT program. The particle-specific volume 0.7300, PBS density at 1.005 and viscosity at 0.01002 as recommended by the SEDFIT program were used. Data were fit at 0.95 confidence level.

### Cell Culture.

All the vitro studies were carried out with BMDMs. BMDMs were first cultured in 10% L929 supernatant, 10% Fetal Bovine Serum (FBS) supplemented DMEM for 5 d and then replaced to DMEM (10% FBS) medium. All the cells were cultured in a humidified 5% CO_2_ incubator at 37 °C. BMDMs were differentiated to M1 or M2 macrophages by treating the cells with either LPS (100 ng/mL) or IL-4 (20 ng/mL), respectively, for 24 h.

### In Vitro Studies.

For internalization studies, either AF647conjugated MANPs or Cy5-TGF si-MANPs were used. The different cell types were treated with 1 µg/mL MANPs for 15 min and then washed with PBS and the cells were collected by gentle scrapping. The cells were then collected by centrifugation and analyzed by flow cytometry. For the antibody-blocking and receptor-blocking experiments, the M2 macrophages were first treated with different concentration of CD206 antibody or monodansyl cadaverine for 30 min and then MANPs were added to the culture for 15 min, and then a similar procedure was followed.

### Mice.

C57BL/6 mice (Jackson Laboratory) were used for all the animal experiments. All the animals were randomly assigned to the experimental groups. Animal studies were approved by the University of Illinois Animal Care and Use Committee. The in vivo PF model was developed by instilling bleomycin (0.015 U/20 g) (54) intratracheally and killed on day 7 of 15 d of instillation. For the adoptive transfer experiment, the recipient mice were instilled with a low dose of bleomycin (0.0075 U/20 g). For the lineage-tracing experiment, Cx3cr1^CreERT2^ (tamoxifen-inducible Cx3cr1 monocyte-specific Cre recombinase) (Jackson Laboratory Cat. no. 021160), Tdtomato^fl/fl^ (Jackson Laboratory Cat. No 007909) mice were crossed to generate inducible Cx3cr1^CreERT2+^; Tdtomato^fl/fl^ mice. Genotyping of mice strains was performed by regular PCR using the recommended primers on the Jackson Laboratory website (https://www.jax.org/). Cx3cr1^CreERT2^ mice were administered tamoxifen (75 mg/kg body weight) via intraperitoneal injection starting before 1 wk of bleomycin instillation and continuing every other day throughout the treatment for Cre induction (17).

### In Vivo Uptake of MANPs.

The internalization of MANPs by the profibrotic macrophages were assessed in the bleomycin instilled mice fibrosis model. The mice (C57Bl6 and Cx3cr1^CreERT2+^ Tdtomato^fl/fl^) were intravenously injected with AF647-MANPs (50 μg/100 µL) 2 h before being killed. The lungs were perfused with PBS and processed for flowcytometry analysis. For comparative analysis of mRNA expression levels of different profibrotic genes in MANP^+^ and MANP^−^ macrophages, the cells were sorted using Cell sorter and then the cells were lysed for further RNA isolation, cDNA isolation, and quantitative PCR.

### Flow Cytometry Analysis.

After the lung tissues collected, they were cut into fine pieces and digested with 1× collagenase in DMEM media for 1 h. The cells were passed through a 100-µm filter, and red blood cells were lysed with lysis buffer. The single cells were stained with CD45-AF700, Gr-1-BV605, CD64-PECy7, SiglecF-BV421, CD11b-AF594, CD206-AF488 (BioLegend). The samples were run on Flow Fortessa (BD Biosciences) and data were analyzed by Kaluza Analysis 2.1 (Beckman). The cells were first gated for live single cells followed by gating strategies required for different cell type. For cell sorting, samples were run on Moflo Astrios and cells were collected in DMEM media until further processed. For biodistribution analysis, the different immune cells were gated following a published protocol (55).

### Adoptive Transfer Experiments.

To test the profibrotic functionality of MANP^+^ macrophages, first bleomycin (0.015 U/20 g) was instilled in C57BL6 mice, and on the 7th day of bleomycin instillation AF647-MANPs were intravenously injected for 2 h. Then, the MANP^+^ as well as MANP^−^ macrophages were sorted by cell sorter. Next 1 × 10^5^ cells were intravenously injected to low-dose bleomycin (0.0075 U/20 g) instilled mice (day 3 of bleomycin instillation). The low dose bleomycin primed mice served as controls. Then, the mice were kept for 7 d and killed for analysis of changes in the macrophage population or inflammation changes. To track the transferred cells, we labeled the sorted cells using CFSE dye before transfer to mice and killed the mice after 3 d of transfer.

### Lung Imaging.

The biodistribution of the MANPs was studied by intravenous injection of AF647-MANPs to fibrotic mice. The different organs were collected at 2 h, 5 d, and 10 d after injection. The organs were analyzed ex vivo under an in vivo imaging system (Perkin-Elmer) and the fluorescence of AF647 was analyzed. The images were captured at field of view 13.2 cm.

### BrdU Assay to Assess Proliferation.

Mice were injected with BrdU (Sigma #B5002) 75 mg/kg 24 h intraperitoneally before being killed. After killing, lungs were perfused with PBS and the single-cell suspension was prepared for flow cytometry as stated before. Anti-BrdU antibody (BioLegend) was used for staining according to the manufacturer’s protocol.

### Therapeutic Effects of TGFβ1-siRNA MANPs.

To test therapeutic effect, either scrambled siRNA-loaded MANPs or the TGFβ1-si-MANPs (50 µg/100 µL) were injected intravenously to the fibrotic mice on day 5 and 10 of bleomycin instillation. For 7-d analysis single-dose and for 15-d analysis two doses of the MANPs were injected.

### Lung Function.

Mice were anesthetized on day 15 of bleomycin instillation. The trachea was exposed and 18-gauge canula was inserted into the trachea through a small incision. The canulated mice was attached to the ventilator of the flexiVent system and then lung measurements were done. After recording the baseline measurements, the nebulizer was loaded with 100 μL of saline and then lung measurements were recorded for 3 min. After lung measurements, the mice were killed by cervical dislocation and the lung tissues were processed.

### Histological Analysis.

For histological analysis of lungs of naïve, fibrotic, and treated mice, after perfusion the lung tissues were fixed using 10% buffered formaldehyde and processed for paraffin section. The histological sectioning and staining for H&E, Trichome staining, and immunostaining for α-SMA were done with the help of the Research Histology Core, University of Illinois, Chicago. The quantification was done using ImageJ software. Further, Aperio Image Scope system (Leica) was used to digitize histology slides. The Orbit Image Analysis software (v3.15) was used to quantify histology images by machine learning (54). The object training function was used to classify fibrotic versus normal mass. The percent fibrotic mass was calculated using ImageJ software.

### ELISA.

Lung tissue was homogenized in lysis buffer (PBS 1×, Triton X-100 0.01%, 1× protease inhibitor). Samples were centrifuged at 13,000 rpm for 20 min at 4 °C and the supernatants were collected. The total amount of cytokines was quantified using TGFβ1 and IL-1β ELISA kits (R&D Systems) according to the manufacturer's protocol and normalized to the total content of protein as quantified by Bradford's reagent.

### Hydroxyproline Assay.

To assess the fibrotic burden and the effect of therapy in reducing the fibrotic burden, amount of collagen was quantified by hydroxyproline assay (Sigma) following the manufacturer’s protocol. For this, the complete left lobe of the lung was collected from all experimental mice.

### Statistics.

Statistical significance was analyzed using Prism v9.1.1 (GraphPad Software) with tests as described in the figure legends. All experiments were repeated independently. Sample size and *P* values are all cited in the figures and their captions.

## Supplementary Material

Supplementary File

## Data Availability

All study data are included in the main text and/or *SI Appendix*.
