# Supplementary material for: Nanoparticle targeting of de novo profibrotic macrophages mitigates lung fibrosis
Source: Proc Natl Acad Sci U S A. 2022 Apr 4;119(15):e2121098119. doi: 10.1073/pnas.2121098119 (PMC9169714; doi:10.1073/pnas.2121098119)
Supplement: Supplementary File [file pnas.2121098119.sapp.pdf]

PNAS
www.pnas.org

Supplementary Information for

Nanoparticle Targeting of de novo Pro-fibrotic Macrophages Mitigates Lung Fibrosis

Abhalaxmi Singh [a,b,c], Sreeparna Chakraborty [a,b,d], Sing Wan Wong [a,b,e], Nicole A. Hefner [a,b], Andrew Stuart [a,b,c] Abdul S. Qadir [a,b], Amitabha Mukhopadhyay [a,b], Kurt Bachmaier [a,b,c] Jae-Won Shin [a,b,e], Jalees Rehman [a,b,c] Asrar B. Malik [a,b,c]*

[a] Department of Pharmacology and Regenerative Medicine, The University of Illinois College of Medicine, E403, Medical Sciences Building, M/C 868, 835 S. Wolcott Avenue, Chicago, IL 60612
[b] Center for Lung and Vascular Biology, The University of Illinois College of Medicine, E403, Medical Sciences Building, M/C 868, 835 S. Wolcott Avenue, Chicago, IL 60612
[c] Nano Biotherapeutics Inc., Chicago, IL 60612
[d] Division of Cardiology, Department of Medicine, The University of Illinois College of Medicine, Chicago, IL 60612
[e] Department of Biomedical Engineering, University of Illinois, Chicago, IL 60612.

*Corresponding author: Asrar B. Malik
Email: abmalik@uic.edu

This PDF file includes:

Figures S1 to S6

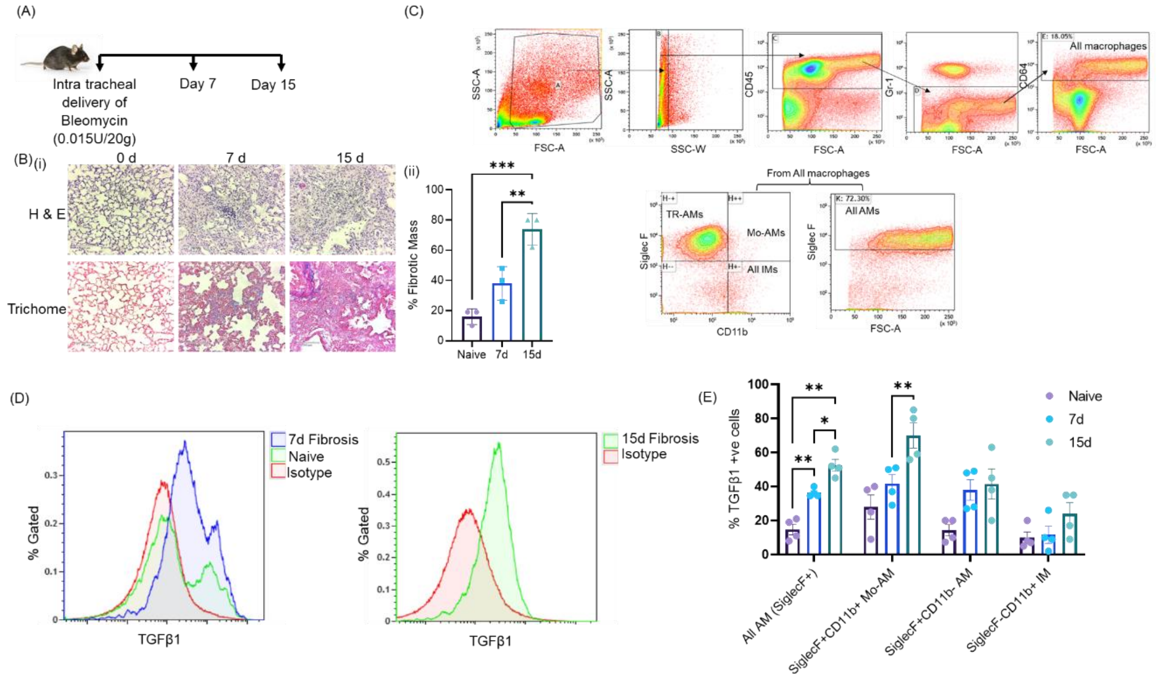

Fig. S1. Changes in lung macrophage population in bleomycin-induced pulmonary fibrosis. (A) Schematic representation of development of bleomycin-induced pulmonary fibrosis mouse model. (B) Histological images of lung at different days of fibrosis induction as assessed by H& E and Trichome staining. (i) Histological images (ii) Quantification of fibrotic mass using ImageJ software. (C) Flow cytometry gating strategy used to analyze macrophage populations. (D) Changes in TGFβ1 expression by all AMs. (Gating strategy: CD45+Gr1-CD64+SglecF+). (E) Changes in TGFβ1 positive population in different lung macrophages after 7d and 15d post bleomycin. Statistical analysis was made by Two-way ANOVA. *<0.05 and ** p<0.005.

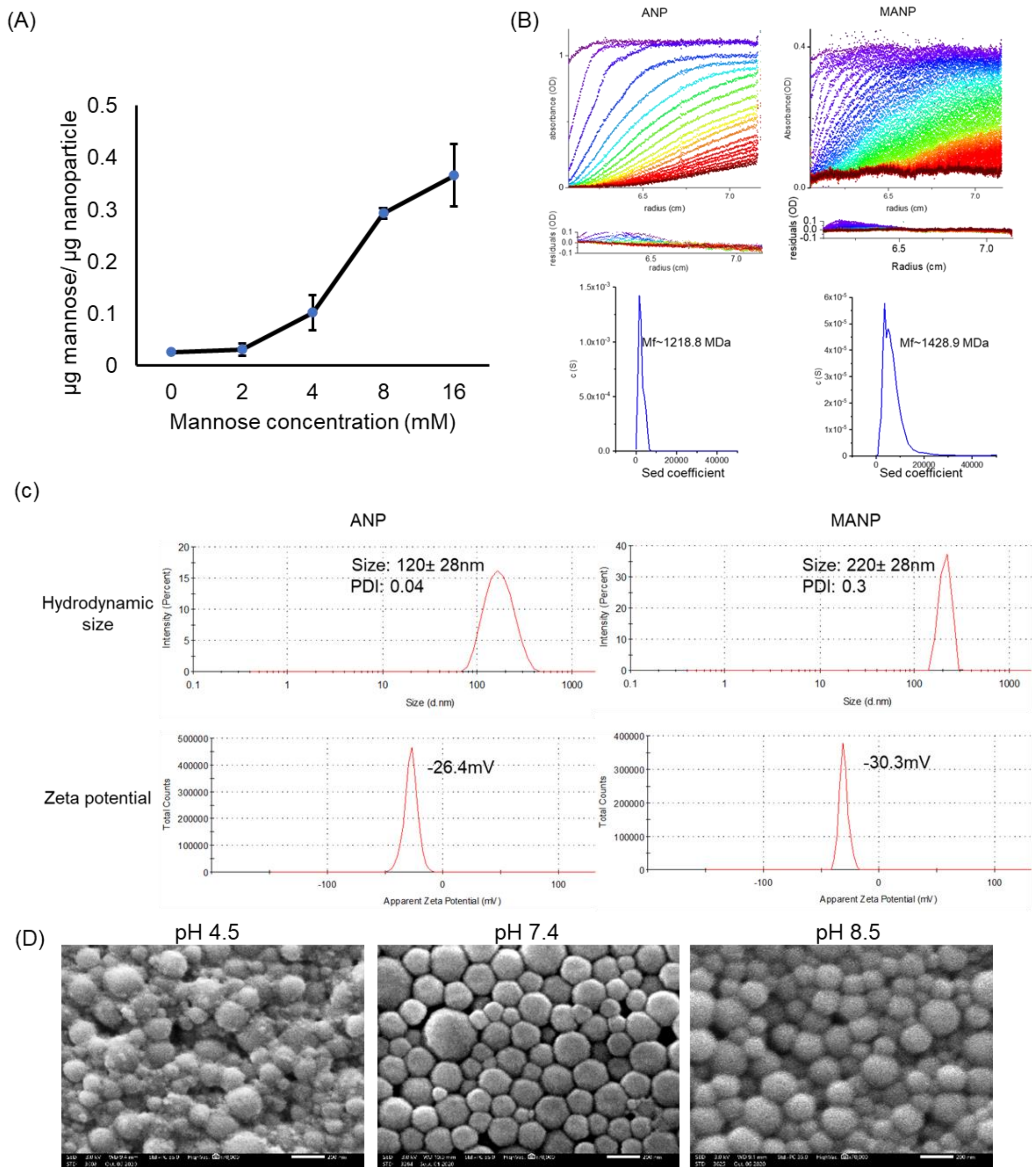

Fig. S2. Physical characteristics of nanoparticles. (A) Quantification of mannose concentration per µg nanoparticle. Three different batches were analyzed. (B) Ultracentrifugation showing sedimentation coefficient and molecular weight changes of nanoparticles before and after mannosylation. (C) Hydrodynamic size and zeta potential of nanoparticles before and after mannosylation as determined by dynamic light scattering. (D) SEM images of MANPs at different pH levels incubated overnight (Scale bar: 200nm).

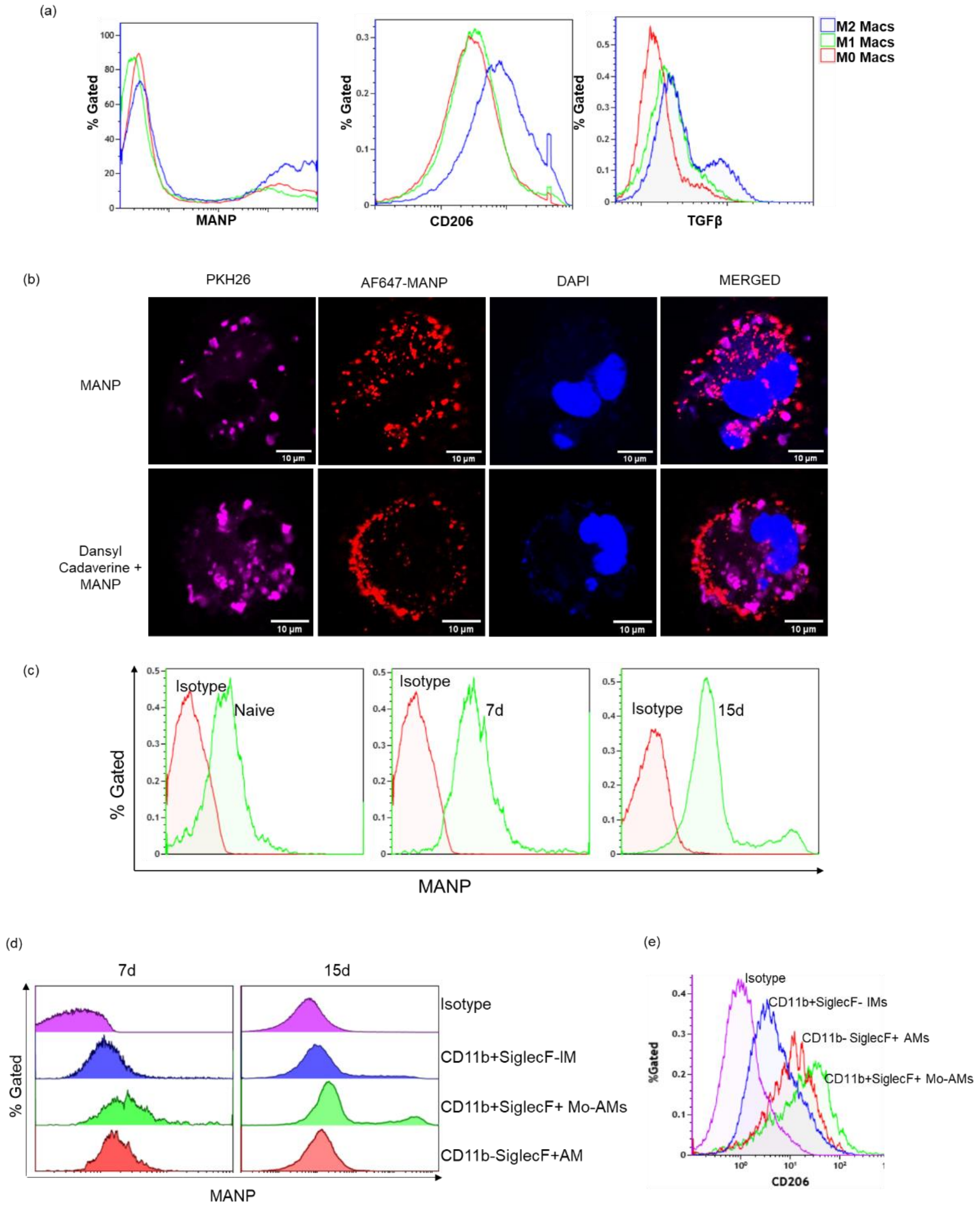

Fig. S3. Macrophages internalize MANPs. (A) Flow cytometry histograms showing M2 macrophages with higher CD206 and TGFβ1 expression and higher MANP internalization (B) Monodansyl cadaverine, an inhibitor of receptor-mediated endocytosis prevented MANP internalization by M2 macrophages as shown by confocal microscopy along with membrane staining with PKH26. (C) Representative flow cytometry histograms for Fig. 2G showing internalization of MANPs by SiglecF+ AMs. In naïve and after 7d and 15 d post bleomycin treated mice (D) Representative flow cytometry histograms for Fig. 2H showing internalization of MANPs by different macrophage population after 7d and 15d post bleomycin treatment. (E) Expression of CD206 in distinct macrophage populations at 15d post bleomycin treatment.

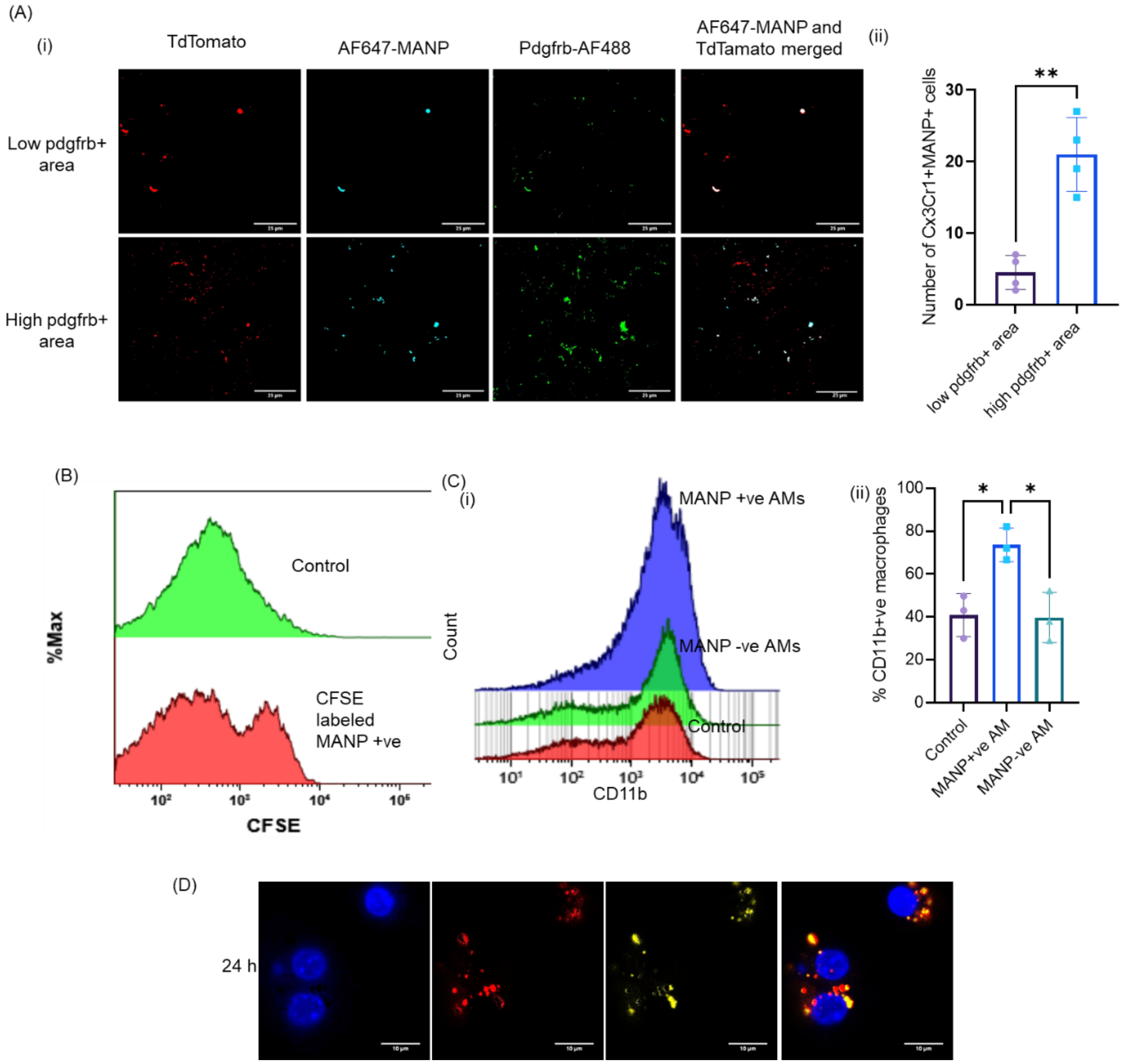

Fig. S4. Localization of MANP+ Mo-AMs and engraftment of adoptively transferred macrophages. (A) Lung immunofluorescence at 15d after bleomycin in Cx3cr1CreERT2+,Tdtomatofl/fl mice. The mice were injected with AF647-MANPs 2h before sacrifice and tissue sections were stained with anti-Pdgfrb antibody. The results show the presence of MANP+TdTomato+ cells in the vicinity of Pdgfrb+ fibroblasts (i) Confocal microscopy images, (ii) Quantification. (B) Flow cytometry evaluation of engraftment of CFSE labeled Mo-AMs in lung after 3 days of injection. (C) CD11b positive macrophages (CD45+Gr1-CD64+) in different groups showing extent of profibrotic effect from adoptively transferred macrophages after 7 day of injection (i) Overlayed flow cytomery plots, (ii) Quantification from 3 independent experiments. (D) Confocal microscopy images show the presence of Cy5-labeled TGFβ1 siRNA from MANPs even at 24h posttreatment. Statistical analysis was carried out by one-way ANOVA and unpaired t-test. *$p<0.05$, ** $p<0.005$.

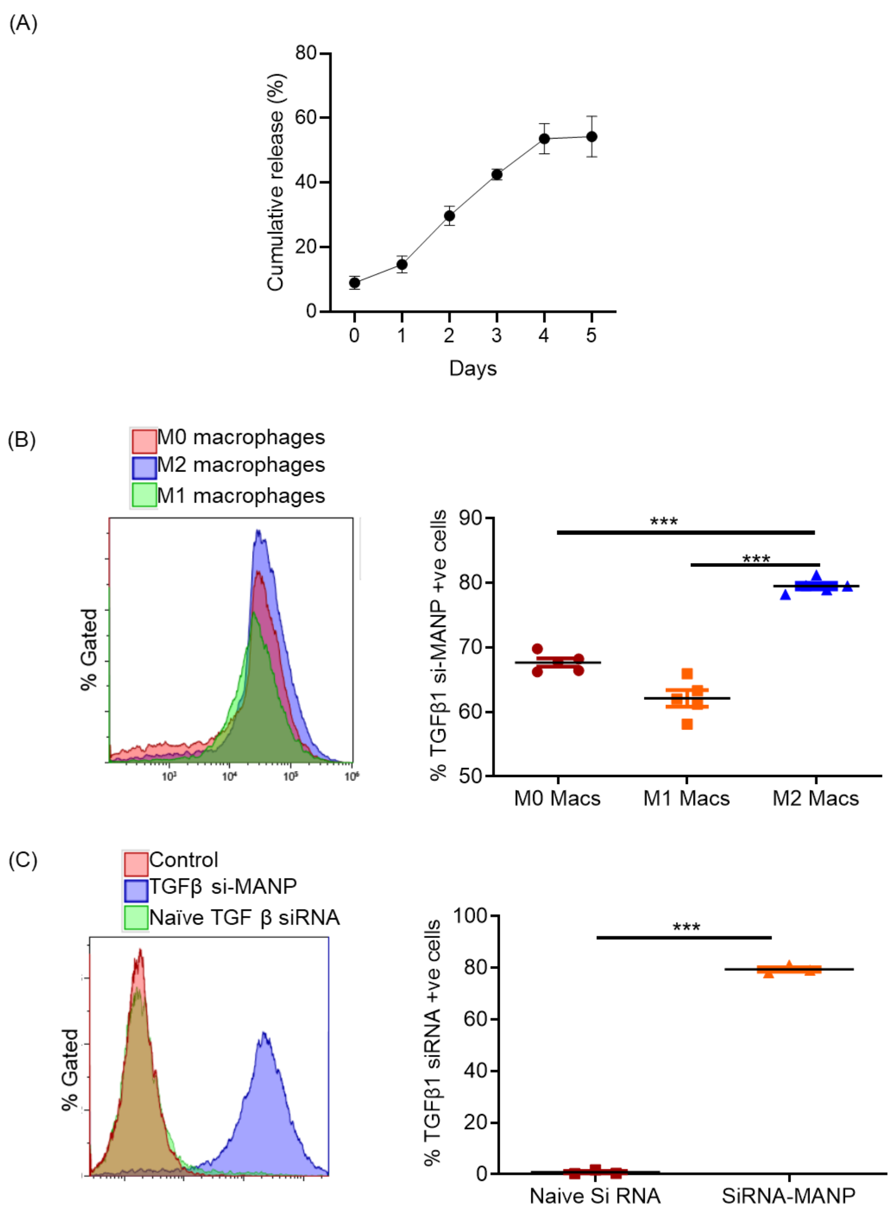

Fig. S5. ***In vitro*** **uptake of Cy5-labeled TGFβ1 siRNA-loaded MANPs.** (A) Release kinetics of Cy5-labeled TGFβ1 siRNA from MANPs. Cy5-TGFβ1 si-MANPs were dispersed in PBS and kept at 37 ℃ in an incubator and the supernatants were collected at different time intervals (B0 Internalization of TGFβ1 si-MANPs by bone marrow derived M0, M1, and M2 macrophages determined by flow cytometry. Internalization was measured 2h after MANP addition to macrophages. (C) Internalization of naïve siRNA vs. siRNA loaded in MANPs in bone marrow derived M2 macrophages. Statistical analysis was carried out by One-way ANOVA. *** p<0.0005.

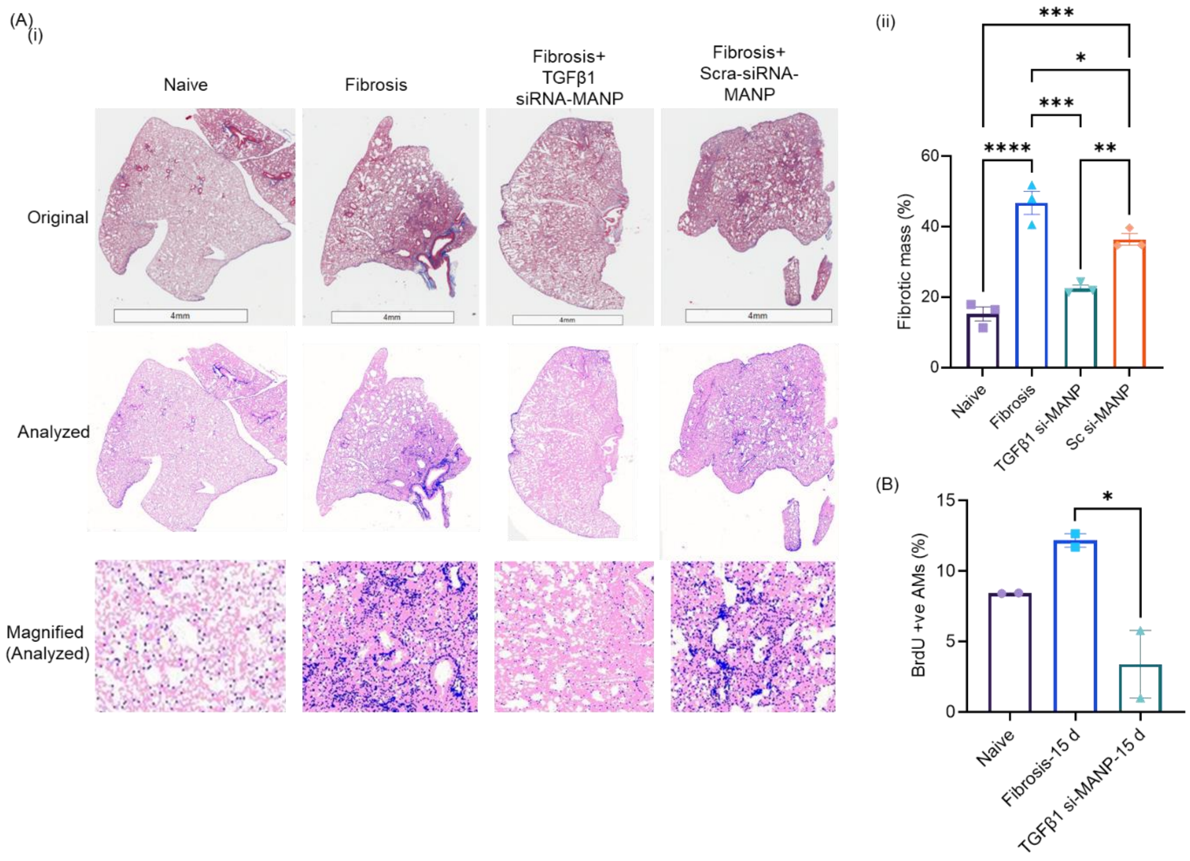

Fig. S6. Therapeutic efficacy of TGFβ1 siRNA-loaded MANPs. (A) Histology of whole section of lungs from naïve mice, mice at Day 15 post-fibrosis induced by bleomycin, and MANP treated mice at Day 15 post-fibrosis induced by bleomycin. The images were analyzed by Orbit Image analysis and ImageJ and the fibrotic mass was calculated in whole lung sections from 3 independent experiments. (i) Trichome staining images, (ii) Quantification of fibrotic mass. (B) BrdU staining showing augmented proliferation of all AMs in fibrotic lung as compared to naïve lung and lung treated with TGFβ1 siRNA-MANP 15d treatment. The latter inhibited proliferation of Mo-AMs. Statistical analysis was carried out by one-way ANOVA. *$p<0.05$, **$p<0.01$, *** $p<0.001$, ****$p<0.0001$.
